# Investigation of Interaction between Dislocation Loop and Coherent Twin Boundary in BCC Ta Film during Nanoindentation

**DOI:** 10.3390/nano7110375

**Published:** 2017-11-06

**Authors:** Cheng Huang, Xianghe Peng, Bo Yang, Yinbo Zhao, Shayuan Weng, Tao Fu

**Affiliations:** 1 College of Aerospace Engineering, Chongqing University, Chongqing 400044, China; huangcheng@cqu.edu.cn (C.H.); yangbo16@cqu.edu.cn (B.Y.); yinbo.zhao@qut.edu.au (Y.Z.); shayweng@foxmail.com (S.W.); 2 State Key Laboratory of Coal Mine Disaster Dynamics and Control, Chongqing University, Chongqing 400044, China

**Keywords:** nanotwinned Ta film, nanoindentation, dislocation loop, deformation mechanisms, molecular dynamics

## Abstract

In this work, the interaction between dislocation loop (DL) and coherent twin boundary (CTB) in a body-centered cubic (BCC) tantalum (Ta) film during nanoindentation was investigated with molecular dynamics (MD) simulation. The formation and propagation of <111> full DLs in the nanotwinned (nt) Ta film during the indentation was observed, and it was found that CTB can strongly affect the stress distribution in the Ta film, and thus change the motion and type of dislocations. There are three kinds of mechanisms for the interaction between DL and CTB in a twinned BCC Ta film: (i) dislocation absorption, (ii) dislocation desorption, and (iii) direct slip transmission. The nucleation of twin boundary dislocations and the formation of the steps in CTB were also observed during the indentation. The mechanisms presented in this work can provide atomic images for understanding the plastic deformation of BCC metals with mirror-symmetry grain boundary structures, and provide available information for the evaluation and design of high-performance nt BCC metallic thin film coatings.

## 1. Introduction

Metallic thin film coatings have attracted considerable attention due to their unique physical, mechanical and thermal properties [1,2,3], such as high strength, high hardness and high melting point, etc. Due to these excellent properties, metallic thin film coatings have been widely used in modern industry. The design, evaluation and application of the thin film coatings require a detailed characterization of their mechanical properties, such as hardness and strength, as well as the underlying deformation mechanisms. Nanoindentation test is an effective method to characterize the mechanical properties of materials, especially suitable for small volumes of material or tiny components. In the existing researches of the nanoindentation for the mechanical properties and plasticity of metallic thin film coatings, attention was mainly paid to face-centered cubic (FCC) metals [3,4,5,6,7]. For example, Fu et al. [3,6] analyzed the effects of coherent twin interfaces on the mechanical properties and hardening behavior of Cu/Ni multilayers under nanoindentation. Zhou et al. [4] investigated the effects of hydrogen charging on the mechanical response of FCC Ni and Pd under nanoindentation. Less progress related to the responses and plastic deformation of body-centered cubic (BCC) metals (e.g., Tantalum (Ta)) subjected to nanoindentation can be found in the literature. Recently, some researchers [8,9,10] performed the nanoindentation on single crystal Ta using molecular dynamics (MD) simulations, in which they found the formation of dislocation loops (DLs) and clarified the plastic deformation of Ta.

It is known that grain boundaries (GBs) may play a crucial role in the mechanical properties and plastic deformations of nanocrystalline materials. These planar defects can act as sinks and sources of dislocations, and hinder the motion of dislocations [11,12]. Coherent twin boundary (CTB) can be regarded as one of the most common GBs in NC materials, which has the lowest GB energy in most cases and can greatly improve the performance of materials, such as strength, hardness and ductility, etc. [13,14,15,16,17]. Recently, the nucleation of DLs on the CTB of nanotwinned (nt) Ta under shear stress was studied by Sandoval et al. using MD simulations [18], but how a DL interacts with the CTB in the nt BCC metal is still unclear. Thus, it should be essential and significant to explore the underlying mechanisms of the interaction between incident DL and CTB to understand the plastic deformation of the BCC metals with mirror-symmetry GBs. Since it has been proven that nanoindentation is a suitable means to investigate the plastic deformations of nano-materials [19,20,21], in this article, we will perform nanoindentation on nt-Ta films with MD simulation to gain an insight into the DL-CTB interaction. To shed light on the interaction between indentation-induced DLs and CTB, we assume that the simulation model is initially defect-free in this work. In Section 2, a detailed simulation approach is introduced. In Section 3, we present and discuss the results obtained in the simulation, and try to identify the interaction mechanisms. Conclusions are drawn in Section 4.

## 2. Methods

**Figure 1:**
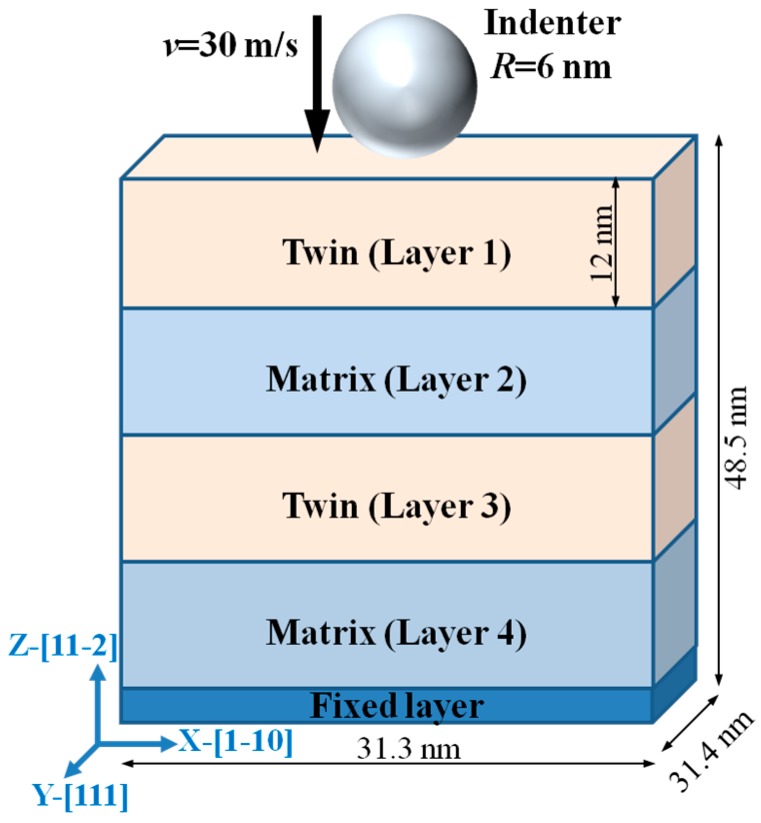
Setup of nanoindentation.

The embedded atom method (EAM) developed by Daw et al. [22] has been widely adopted to study the defects and their evolution [23], dislocation-CTB interaction [13,24,25], and deposition and growth problems [26] in metals and alloys. In the MD simulation for the nanoindentation on nt-Ta films, EAM potential [22] is employed and the parameters suggested by Ravelo et al. [27] are adopted. The potential can well describe the deformation of Ta under nanoindentation [10] and reproduce the tilted GBs and twin structures in Ta [28]. The setup for the simulation is shown in Figure 1, which contains a sample and a fictitious indenter. The size of the sample is 31.3 nm, 31.4 nm and 48.5 nm in *x*, *y* and *z* directions, and the *x*, *y* and *z* directions correspond to the lattice orientations of $\left[1\bar{1}0\right]$, [111] and $\left[11\bar{2}\right]$, respectively. The sample contains three CTBs, and the thickness of each twin or matrix layer is 12 nm. During the indentation, the atoms in the bottom three layers are fixed as boundary atoms to prevent the sample from shifting, and the atoms in the rest layers are kept at a constant temperature with a Langevin thermostat [29] as thermostat atoms. The motion of the thermostat atoms follows the classical Newton’s second law, and hence these atoms are called Newtonian atoms. Periodic boundary conditions are imposed in both *x* and *y* directions. The time step is set as 1 fs in the simulation. Before indentation, the conjugate gradient (CG) algorithm is applied to optimize the structure. Then, the sample is relaxed at 10 K using a Nose-Hoover (NPT) thermostat for 40 ps to reach a thermal equilibrium state. To our knowledge [30,31,32,33,34], the relaxation time of 40 ps is sufficiently long for a sample to an equilibration state. For example, Fu et al. [33] relaxed the nanotwinned vanadium nitride at *T* = 300 K for 30 ps. As the time step for MD simulations is of fs, high indentation speed of 10–100 m/s is commonly used to perform MD nanoindentation simulations [35,36,37,38,39]. For example, Zhang et al. [38] perform MD nanoindentation with the speed of 100 m/s. Gao et al. [39] studied the influence of the indentation speed on the results in their simulations of nanoindentation on Fe using the indentation speed spanning from 10 to 100 m/s, and found no significant influence on the main results. It should be noted that in the simulation for the nanoindentation on Ta with the speed spanning from 3.4 to 34 m/s [10] showed that the variation of the indentation speed in this range has insignificant influence on the results. Inspired by the above work, we choose the indentation speed of 30 m/s in our simulation for the nanoindentation, which is performed at 10 K with a fictitious spherical indenter of 60 Å, and the indentation time is 180 ps, and the maximum indentation depth is 52 Å, which is enough for studying the interaction between twin boundary and indentation induced dislocations. 

The code OVITO [40] is employed to visualize the simulation results. Crystal defects are identified with the polyhedral template matching (PTM) [41] and the dislocation extraction algorithm (DXA) [42], of which PTM can identify simple local lattice structures (FCC, BCC, HCP, etc.), and DXA can identify dislocations, determine their Burgers vectors, and output dislocation lines. In the following, BCC, FCC and hexagonal close-packed (HCP) structures will be indicated with blue, green and red, respectively; the <111> type and <100> type dislocations will be indicated with green and pink lines, respectively, and Burger's vectors will be represented with blue arrows.

## 3. Results and Discussion

**Figure 2:**
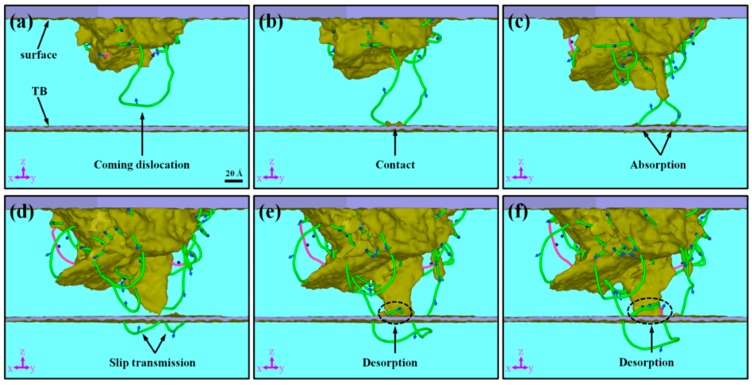
Evolution of dislocations in sample: (**a**) *h* = 23 Å, (**b**) *h* = 27.05 Å, (**c**) *h* = 34.1 Å, (**d**) *h* = 43.4 Å, (**e**) *h* = 45.95 Å, and (**f**) *h* = 47.45 Å. <111> type dislocations are indicated with green lines, <100> type dislocations with pink lines, and Burger’s vectors with blue arrows.

Figure 2 shows the evolution of the microstructure in the sample under indentation, detected with DXA, where it can be seen that <111> DLs form beneath the indenter, grow and propagate during the progress of the indentation, which is consistent with that reported by Goel et al. [8] and Remington et al. [8,9]. This kind of DL is different from that in FCC metals. As we know, the DL in a FCC metal consists of two partial dislocations separated by a stacking fault, while that in the BCC metal is only a full <111> dislocation. This difference may lead to different mechanisms of DL-CTB interaction.

It can be seen in Figure 2a that with the increase of the indentation depth, *h*, a DL moves along the <111> direction towards the CTB, and then contacts the CTB (Figure 2b). In Figure 2b the CTB acts as a barrier that hinders the expansion of the DL. With the further increase of *h*, more DLs moving towards the CTB are obstructed by and interact with the CTB, as shown in Figure 2c–f. In Figure 2c, the <111> DL is absorbed into the CTB, which, together with Figure 2a,b, can generally be regarded as dislocation absorption by CTB. With the continuation of indentation, new <111> DLs nucleate from the other side of the CTB, as shown in Figure 2d. The process between Figure 2c,d can be referred to as slip transmission. It is interesting in Figure 2e,f that when two DLs contact and are absorbed by the CTB, new <111> and <001> dislocations nucleate and grow. We call this phenomenon desorption of dislocations. The processes of dislocation absorption, desorption and direct slip transmission in the nt BCC metal films are somewhat similar to that in nt FCC metals [25,43,44], which are generally referred to as slip transfer [45]. 

**Figure 3:**
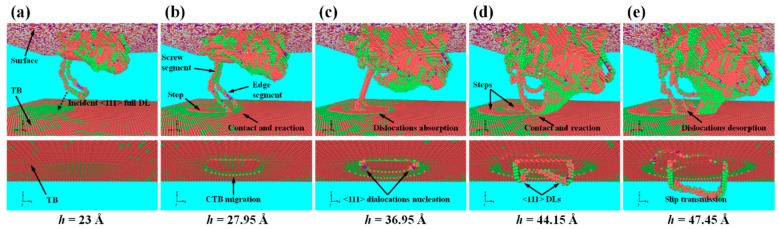
Microstructure evolution of the sample at different *h*, with atoms colored by polyhedral template matching (PTM). Upper row of figures show defects in the first layer, lower row of figures show defects in the second layer.

Figure 3 shows the evolution of the atomic configuration related to DL-CTB interactions in the nt-Ta film under nanoindentation, detected by PTM, where the atoms in BCC structure have been removed for clarity. It can be seen in Figure 3a that an incident <111> full DL, which contains screw and edge components, moves towards the CTB. Although it has not yet touched the CTB, the change in the local structure of the CTB in the moving direction of the DL can be observed. With the increase of *h*, the edge components contact and react with the CTB, resulting in the absorption of dislocations, the migration of the CTB and the formation of steps (Figure 3b,c), while the screw components merge and annihilate, leading to the pinch-off and release of the DL (see Figure 3c,d). Meanwhile, a new <111> DL nucleates and glides in the lower layer, as shown in Figure 3c–e. With the further increase of *h*, other DLs contact and react with the CTB, resulting in the formation of new steps and the desorption of DLs (Figure 3d,e).

**Figure 4:**
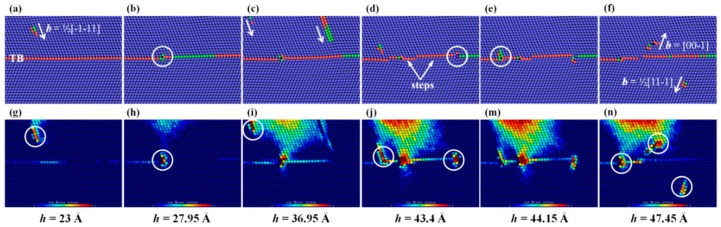
Sliced atomic configurations related to dislocationcoherent twin boundary (CTB) interaction, in (**a**–**f**) atoms are colored by PTM, corresponding distributions of Mises stress are shown in (**g**–**n**). In Figures (**a**)–(**f**) the dislocations activities are indicated by white circles, and the corresponding stress concentration in Figures (**g**)–(**n**) is also indicated by white circles.

To further study the dislocations-CTB interaction, we show in Figure 4 the sliced atomic configurations on the $\left(\bar{1}10\right)$ planes of the sample during nanoindentation. In Figure 4a–f, the absorption, desorption and direct slip transmission of dislocations can be observed clearly. At first, a dislocation with $b=1/2[\bar{1}\bar{1}1]$ moves towards the CTB, as shown in Figure 4a. Then, the dislocations approaching the CTB are blocked and react with the CTB. As can be seen in Figure 4b (*h* = 27.95 Å), the blocked dislocation is absorbed by the CTB, resulting in the migration of a segment of the CTB and the formation of a step. It should be noted that at this moment, the dislocation is stored in the CTB, and does not glide into the lower layer. When *h* = 36.95 Å (Figure 4c), the other two incident dislocations approach the CTB, and the process from Figure 4a,b will be repeated, resulting in the formation of new steps, as shown in Figure 4c,d. With the increase of *h*, two new dislocations nucleate from the CTB, of which the one with $b=1/2[11\bar{1}]$ glides into the second layer, while the other with $b=[00\bar{1}]$ moves into the first layer, as shown in Figure 4e,f, noting that the interaction is accompanied by the nucleation of a 1/6<111> twin boundary dislocation (TBD), which, to our knowledge, is a common type of dislocation in BCC metals [46].

To penetrate through the CTB, the applied driving stress on the dislocation should be sufficiently large to overcome the repulsion from the CTB [25]. To further explore the effects of the CTB on the dislocations movement, the corresponding distributions of von Mises equivalent stress are calculated and shown in Figure 4g–n, where it can be seen that the stress level inside the dislocation cores is much higher than that in the defect-free region, which implies that the CTB can strongly affect the distribution of the stress in the nt-Ta film, and thus affect the types and motion of dislocations during nanoindentation.

**Figure 5:**
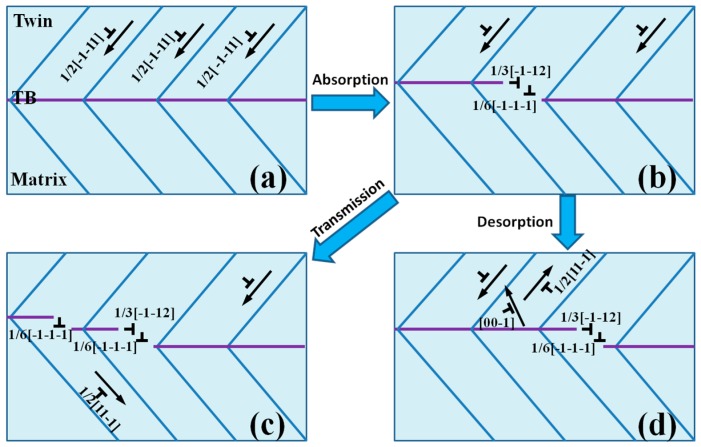
Schematic illustration for interaction between dislocation and CTB. (**a**) a set of incident <111> full dislocations, (**b**) dislocations absorption, (**c**) direct slip transmission, and (**d**) dislocations desorption.

The mechanisms of the interaction between dislocation and the CTB are summarized and shown in Figure 5, where it can be seen that an incident dislocation (Figure 5a) approaching the CTB is forced to enter the CTB. However, it cannot pass through the CTB and get into the lower layer, because CTB is a kind of high-angle GB. Consequently, the dislocation is absorbed by the CTB (see Figure 2c), accompanied by the nucleation of a 1/6<111> TBD, as schematically shown in Figure 5b. This reaction can be summarized as: $1/2[\bar{1}\bar{1}1]\to 1/3[\bar{1}\bar{1}2]+1/6[\bar{1}\bar{1}\bar{1}]$. There are two possible pathways during the following indentation: (1) direct slip transmission of an edge dislocation across the TB (see Figure 2d), leaving behind a TBD with $b=1/6[\bar{1}\bar{1}\bar{1}]$ (see Figure 5c); (2) an edge dislocation cannot pass through, but is absorbed by the TB. Meanwhile, the dislocations stored in the TB are desorbed into the first layer, forming a new dislocation, which slips along $\left[00\bar{1}\right]$ or $\left[11\bar{1}\right]$ direction (Figure 2f), as schematically shown in Figure 5d. And this reaction can be expressed as: $-1/3[\bar{1}\bar{1}2]-1/6[\bar{1}\bar{1}\bar{1}]\to 1/2[11\bar{1}]$ or $-1/2[\bar{1}\bar{1}1]+1/2[\bar{1}\bar{1}\bar{1}]\to [00\bar{1}]$. To summarize, there are three kinds of mechanisms for the interaction between DL and the CTB in the BCC Ta film: (i) absorption of incident dislocations by the CTB, (ii) desorption of the dislocations blocked at the CTB, and (iii) direct slip transmission when incident dislocations pass through the CTB.

## 4. Conclusions

We performed MD simulation for response of a multilayered nano-twinned tantalum film under nanoindentation to shed light on the mechanisms of the interaction between dislocation loop (DL) and coherent twin boundary (CTB). We observed the formation and propagation of <111> full DLs in the nt-Ta film during the nanoindentation, and concluded the following mechanisms: (i) the absorption of the incident dislocations by the CTB, (ii) the desorption of the dislocations blocked at the CTB, (iii) direct slip transmission when incident dislocations pass through the CTB. It was also found that the CTB can strongly affect the distribution of the stress in the Ta film, and thus affect the types and motion of dislocations during nanoindentation. We schematically illustrated the mechanisms of the interaction between DL and CTB, including the formation of the steps in the CTB and the nucleation of TBDs.

It is well established that the mechanical properties of nanotwinned metals are closely related to the interaction between dislocations and CTB [47,48], but many details of this interaction have not yet been well understood in BCC metals. It is noteworthy that the interaction between dislocation and CTB in nt BCC metals may play an important role in the plastic deformation of materials. Therefore, the findings in this work could contribute to the understanding of the underlying interaction between DLs and CTB in nt BCC metals, and help to understand the plasticity, and design the microstructure of BCC metallic films for better performance, such as high hardness and strength.

## References

[1] Varillas J., Očenášek J., Torner J., Alcalá J. (2017). Unraveling deformation mechanisms around FCC and BCC nanocontacts through slip trace and pileup topography analyses. Acta Mater..

[2] Mohr M., Daccache L., Horvat S., Brühne K., Jacob T., Fecht H.-J. (2017). Influence of grain boundaries on elasticity and thermal conductivity of nanocrystalline diamond films. Acta Mater..

[3] Fu T., Peng X., Chen X., Weng S., Hu N., Li Q., Wang Z. (2016). Molecular dynamics simulation of nanoindentation on Cu/Ni nanotwinned multilayer films using a spherical indenter. Sci. Rep..

[4] Zhou X., Ouyang B., Curtin W.A., Song J. (2016). Atomistic investigation of the influence of hydrogen on dislocation nucleation during nanoindentation in Ni and Pd. Acta Mater..

[5] Liu Q., Deng L., Wang X. (2016). Interactions between prismatic dislocation loop and coherent twin boundary under nanoindentation investigated by molecular dynamics. Mater. Sci. Eng. A.

[6] Fu T., Peng X., Weng S., Zhao Y., Gao F., Deng L., Wang Z. (2016). Molecular dynamics simulation of effects of twin interfaces on Cu/Ni multilayers. Mater. Sci. Eng. A.

[7] Li J., Fang Q.H., Liu B., Liu Y., Liu Y.W., Wen P.H. (2015). Mechanism of crack healing at room temperature revealed by atomistic simulations. Acta Mater..

[8] Goel S., Beake B., Chan C.-W., Haque Faisal N., Dunne N. (2015). Twinning anisotropy of tantalum during nanoindentation. Mater. Sci. Eng. A.

[9] Remington T.P., Ruestes C.J., Bringa E.M., Remington B.A., Lu C.H., Kad B., Meyers M.A. (2014). Plastic deformation in nanoindentation of tantalum: A new mechanism for prismatic loop formation. Acta Mater..

[10] Ruestes C.J., Stukowski A., Tang Y., Tramontina D.R., Erhart P., Remington B.A., Urbassek H.M., Meyers M.A., Bringa E.M. (2014). Atomistic simulation of tantalum nanoindentation: Effects of indenter diameter, penetration velocity, and interatomic potentials on defect mechanisms and evolution. Mater. Sci. Eng. A.

[11] Hahn E.N., Meyers M.A. (2015). Grain-size dependent mechanical behavior of nanocrystalline metals. Mater. Sci. Eng. A.

[12] Meyers M.A., Mishra A., Benson D.J. (2006). Mechanical properties of nanocrystalline materials. Prog. Mater. Sci..

[13] Li X., Wei Y., Lu L., Lu K., Gao H. (2010). Dislocation nucleation governed softening and maximum strength in nano-twinned metals. Nature.

[14] Lu L., Shen Y., Chen X., Qian L., Lu K. (2004). Ultrahigh strength and high electrical conductivity in copper. Science.

[15] Lu L., Chen X., Huang X., Lu K. (2009). Revealing the Maximum Strength in Nanotwinned Copper. Nature.

[16] Tian Y., Xu B., Yu D., Ma Y., Wang Y., Jiang Y., Hu W., Tang C., Gao Y., Luo K. (2013). Ultrahard nanotwinned cubic boron nitride. Nature.

[17] Huang Q., Yu D., Xu B., Hu W., Ma Y., Wang Y., Zhao Z., Wen B., He J., Liu Z. (2014). Nanotwinned diamond with unprecedented hardness and stability. Nature.

[18] Sandoval L.A., Surh M.P., Chernov A.A., Richards D.F. (2013). Growth of deformation twins in tantalum via coherent twin boundary migration. J. Appl. Phys..

[19] Li J., Guo J., Luo H., Fang Q., Wu H., Zhang L., Liu Y. (2016). Study of nanoindentation mechanical response of nanocrystalline structures using molecular dynamics simulations. Appl. Surf. Sci..

[20] Sha Z.D., Branicio P.S., Sorkin V., Pei Q.X., Zhang Y.W. (2011). Effects of grain size and temperature on mechanical and failure properties of ultrananocrystalline diamond. Diam. Relat. Mater..

[21] Sha Z.D., Quek S.S., Pei Q.X., Liu Z.S., Wang T.J., Shenoy V.B., Zhang Y.W. (2014). Inverse pseudo Hall-Petch relation in polycrystalline graphene. Sci. Rep..

[22] Daw M.S., Baskes M.I. (1984). Embedded-atom method: Derivation and application to impurities, surfaces, and other defects in metals. Phys. Rev. B.

[23] Zhou H., Gao H. (2015). A Plastic Deformation Mechanism by Necklace Dislocations Near Crack-like Defects in Nanotwinned Metals. J. Appl. Mech..

[24] Zhu Y., Li Z., Huang M., Liu Y. (2015). Strengthening mechanisms of the nanolayered polycrystalline metallic multilayers assisted by twins. Int. J. Plast..

[25] Jin Z.H., Gumbsch P., Albe K., Ma E., Lu K., Gleiter H., Hahn H. (2008). Interactions between non-screw lattice dislocations and coherent twin boundaries in face-centered cubic metals. Acta Mater..

[26] Fu T., Peng X., Zhao Y., Feng C., Huang C., Li Q., Wang Z. (2016). MD simulation of effect of crystal orientations and substrate temperature on growth of Cu/Ni bilayer films. Appl. Phys. A.

[27] Ravelo R., Germann T.C., Guerrero O., An Q., Holian B.L. (2013). Shock-induced plasticity in tantalum single crystals: Interatomic potentials and large-scale molecular-dynamics simulations. Phys. Rev. B.

[28] Hahn E.N., Fensin S.J., Germann T.C., Meyers M.A. (2016). Symmetric tilt boundaries in body-centered cubic tantalum. Scr. Mater..

[29] Adelman S.A. (1976). Generalized Langevin equation approach for atom/solid-surface scattering: General formulation for classical scattering off harmonic solids. J. Chem. Phys..

[30] Wu Z.X., Zhang Y.W., Srolovitz D.J. (2009). Dislocation–twin interaction mechanisms for ultrahigh strength and ductility in nanotwinned metals. Acta Mater..

[31] Salehinia I., Lawrence S.K., Bahr D.F. (2013). The effect of crystal orientation on the stochastic behavior of dislocation nucleation and multiplication during nanoindentation. Acta Mater..

[32] Fu T., Peng X., Zhao Y., Sun R., Yin D., Hu N., Wang Z. (2015). Molecular dynamics simulation of the slip systems in VN. RSC Adv..

[33] Fu T., Peng X., Huang C., Zhao Y., Weng S., Chen X., Hu N. (2017). Effects of twin boundaries in vanadium nitride films subjected to tensile/compressive deformations. Appl. Surf. Sci..

[34] Aghababaei R., Joshi S.P. (2014). Micromechanics of tensile twinning in magnesium gleaned from molecular dynamics simulations. Acta Mater..

[35] Alhafez I.A., Ruestes C.J., Gao Y., Urbassek H.M. (2016). Nanoindentation of hcp metals: A comparative simulation study of the evolution of dislocation networks. Nanotechnology.

[36] Yang B., Zheng B., Hu X., Zhang K., Li Y., He P., Yue Z. (2016). Atomistic simulation of nanoindentation on incipient plasticity and dislocation evolution in γ/γ′ phase with interface and void. Comput. Mater. Sci..

[37] Fang T.-H., Chang W.-Y., Huang J.-J. (2009). Dynamic characteristics of nanoindentation using atomistic simulation. Acta Mater..

[38] Zhang Z., Yang S., Guo D., Yuan B., Guo X., Zhang B., Huo Y. (2015). Deformation twinning evolution from a single crystal in a face-centered-cubic ternary alloy. Sci. Rep..

[39] Gao Y., Ruestes C.J., Tramontina D.R., Urbassek H.M. (2015). Comparative simulation study of the structure of the plastic zone produced by nanoindentation. J. Mech. Phys. Solids.

[40] Stukowski A. (2010). Visualization and analysis of atomistic simulation data with OVITO–the Open Visualization Tool. Model. Simul. Mater. Sci. Eng..

[41] Shi Z., Singh C.V. (2016). Competing twinning mechanisms in body-centered cubic metallic nanowires. Scr. Mater..

[42] Stukowski A., Bulatov V.V., Arsenlis A. (2012). Automated identification and indexing of dislocations in crystal interfaces. Model. Simul. Mater. Sci. Eng..

[43] Sun J., Fang L., Sun K., Han J. (2011). Direct observation of dislocations originating from perfect twin boundaries. Scr. Mater..

[44] Li N., Wang J., Misra A., Zhang X., Huang J.Y., Hirth J.P. (2011). Twinning dislocation multiplication at a coherent twin boundary. Acta Mater..

[45] Zhu T., Gao H. (2012). Plastic deformation mechanism in nanotwinned metals: An insight from molecular dynamics and mechanistic modeling. Scr. Mater..

[46] Bristowe P.D., Crocker A.G. (1975). A computer simulation study of the structures of twin boundaries in body-centred cubic crystals. Philos. Mag..

[47] Zhu L., Ruan H., Li X., Dao M., Gao H., Lu J. (2011). Modeling grain size dependent optimal twin spacing for achieving ultimate high strength and related high ductility in nanotwinned metals. Acta Mater..

[48] Sansoz F., Lu K., Zhu T., Misra A. (2016). Strengthening and plasticity in nanotwinned metals. MRS Bull..

